# Editorial: Current Perspectives in Cognitive Processing by Domesticated Animals

**DOI:** 10.3389/fpsyg.2021.736717

**Published:** 2021-08-23

**Authors:** Katherine Bruce, David A. Leavens, Sarah T. Boysen

**Affiliations:** ^1^Department of Psychology, University of North Carolina at Wilmington, Wilmington, NC, United States; ^2^School of Psychology, University of Sussex, Brighton, United Kingdom; ^3^Center for Animal Welfare Science, Department of Pathobiology, Purdue University, West Lafayette, IN, United States; ^4^Comparative Cognition Project, Sunbury, OH, United States

**Keywords:** animal cognition, animals, non-human cognition, cognitive processing, behavior

Recently, studies of cognitive processing in domestic animals, especially dogs, seem to have increased exponentially, and research with more typical laboratory animals, such as rats, pigeons, and non-human primates seems to be declining. Funding for behavioral and/or cognitive work with animals has always been challenging, and as costs for animal housing, care, and per diems have increased significantly in the past two decades, researchers have looked to other subject pools that do not require major funding for conducting relevant and important studies that can contribute significantly to our field. Thus, companion animals, notably dogs, have become an important resource for studies of animal cognition, as well as other accessible and less-studied species like goats, horses, and pigs, among others. The Research Topic, entitled “Current Perspectives in Cognitive Processing in Domestic Animals,” included 10 papers covering a range of topics and species, with summaries of each paper provided here.

In the initial paper, Csoltova and Mehinagic presented a review and summary of recent research on assessment of dog positive-emotion. They describe a variety of new methodologies, measures, approaches, and techniques looking at the perception, processing, and response assessment in dog positive emotion research. While much past effort has focused on the negative aspects of emotional reactivity and responding by dogs under conditions of duress or fear, these authors provided aspects of positive emotion evaluation of dogs and proposed possible new directions for future research in both short-term and longer-term emotional states assessment in dogs. Finally, the review points out potential limitations and needs in current research methods. In a second paper using dog subjects, Kiss et al., the investigators explored how dogs' attention was affected by their owners attention or inattention to their performance during a fetching task with an unfamiliar person. They were concerned whether the dogs were susceptible to the “audience effect,” that is, was their performance affected if they were being watched by their owner or if they were instead ignored. Dogs' performance and behavioral responses were recorded, and these data were subsequently complemented with the dogs' spectral EEG sleep profile which was recorded during a 3-h daytime nap that the dogs took in the laboratory. The results indicated a relationship between the individual dog's susceptibility to the audience effect and the spectral power of REM and non-REM sleep. Both sets of findings provide support for dogs' human-like susceptibility to the audience effect, and how such a trait may be linked to more complex mechanisms like reputation management.

A third paper that presented findings from dogs also provided comparative data from captive born wolves, to look for similarities and differences between the two species on a quantity discrimination task. Numerical skills have been examined in a wide range of species to date, from mammals like non-human primates to birds, fish, even salamanders, and evidence for rudimentary to sophisticated numerical competence have been demonstrated across a variety of tasks. In this study, Rivas-Blanco et al. compared the ability of dogs and wolves to recognize either the larger or small of two arrays presented simultaneously as an array of dots on a touchscreen, computer-interfaced system. The apparatus allowed both canid subjects to make their selection on glass panes in front of the screen, using their muzzles, and the pressure-measuring sensors were then activated and linked to a computer which controlled the trials, subjects' data, and activated a remote reward dispenser. Stimulus pairs in the small range included 1–8 dots in an array and combinations of larger numbers (8–32 dots). Arrays were controlled for cumulative surface area, size, and position on the screen. Results indicated that dogs were able to discriminate between two numbers, and their performance was worse as the ratio between the numbers increased, thus conforming to Weber's Law. However, non-numerical variables like dot size did have an impact on performance. In a second study, hand-reared wolves completed the same task, and they, too, were able to distinguish between two quantities of increasing ratios, and performance also both species. It was not possible to determine whether two distinct number processing systems were operating in either species, as have been speculated for other species similarly tested. In both species tested here, they may have used non-numerical cues, when possible, as well as the numerical information, to solve the task. Overall, both studies provide ample evidence that both dogs and wolves can readily distinguish between quantities of varying ratios and magnitudes but will depend upon non-numerical cues if available. These studies also provide insights and suggestions for future research comparing dogs and wolves on quantity judgments and other cognitive phenomena.

In the next paper focusing on dog subjects, Savalli and Mariti present a thoughtful review of current ideas about the role of caregivers and their dogs, using the term “tutor” to represent the human caretaker, either child or adult, and the relationship between the two species. Clearly the potential for a strong bond exists in dogs and humans, as, as the authors explore, Bowlby's Attachment Theory provides an intriguing mélange of theoretical perspectives and possibilities for the emergence of these bonds, and their impact on both the tutor and the dog. They also suggest that Attachment Theory alone is insufficient to account for the range of two-way interactions between dog and tutor, and proposed that Friendship Theory, while typically not applied to non-human animals, might bridge the gap for explaining the depth and range of bonding that occurs between us and our dogs, or that which emerges as a child grows up with a dog companion. Similarly, relationships between conspecifics, as observed in other species like primate, are also discussed, including the early relationships between female dogs and their offspring, and later relationships between adult dogs that live in proximity to one another. Clearly the dog-tutor relationship is a complex one, and Savalli and Mariti remind us that dogs, like humans, possess attachment and caregiving systems, and as such, offer opportunities for new directions in exploring the complexities of these systems in both dogs and humans.

Four of the articles in this Research Topic focus on the cognitive abilities of domesticated species other than dogs. Croney and Boysen describe an innovative set of procedures to train two Panepinto micro pigs and two Yorkshire pigs (*Sus scrofa*) to use a joystick to respond to visual images on a computer screen. Using an adaptation of the SIDE task that has been used with rhesus monkeys, Croney and Boysen found that the pigs showed impressive motor dexterity to acquire this task. In addition, they remind us of critical methodological considerations for the study of cognitive abilities in other species, including visual (location of computer screen) and motor (manipulation of the joystick) adaptations that must be assessed prior to testing.

Trosch et al. also emphasize the importance of procedural modifications in data collection in their work with Welsh ponies, *Equus caballus*. In their study on object permanence in horses, they also made several elegant procedural adjustments to rule out alternative interpretations related to specific behaviors of the subject species (e.g., the Clever Hans effect). Across two experiments, they showed that these ponies exhibited Stage 5a object permanence, that is, retrieval of an object that had been hidden in two or three locations (visible displacement). In addition, the findings of both the Croney and Boysen and Trosch et al. studies highlight the applications of cognitive processing studies to improving animal welfare.

Lansade et al. also studied Welsh ponies but focused on implications of cognitive processing related to specific interactions with humans. In a set of studies on horses' recognition related to humans, Lansade et al. showed that these ponies discriminated familiar and unfamiliar photographs of human faces even when salient features of the faces were altered (e.g., hair length or color, visibility of eyes, facial orientation). Further, they reported some preference for these familiar, but previously unencountered, humans in social tests. These data underscore the impact of the coevolution of humans and horses showing the extraordinary attention to and discrimination of human features by some domesticated species.

Nawroth et al. reported that goats (*Capra hircus*) can use some human pointing cues to identify containers that contain food rewards, in a version of the Object Choice Task (OCT). In this protocol, one of several containers was covertly baited with a reward, and an experimenter provided a pointing gesture to the baited container. Twenty goats were recruited for the study and administered a pre-test to determine whether they would follow a pointing gesture to the baited bucket for six consecutive trials: nine goats reached this criterion. These goats were tested with three different pointing gestures—proximal (sitting between the two buckets, pointing to the baited bucket about 30 cm. from the pointing finger, crossed (sitting between the two buckets, pointing to the baited bucket across the experimenter's body, about 48 cm. from the pointing), and asymmetrical (sitting behind one bucket and pointing to the other bucket at about 90 cm.). These experimental conditions were compared to a control condition in which the experimenter displayed no pointing cue. The goats performed better when the end of the digit was relatively close to the target container (proximal and crossed), compared to the asymmetrical and control conditions. This demonstration adds to a large and growing literature on the capacity of domesticated animals to follow human communicative cues.

In a series of three studies, Kubinyi et al. explored the influence of owners' affective expressions on dogs' (*Canis lupus familiaris*) fetching and looking behavior. In the first study, twelve dogs were asked to fetch either a toy or a bracelet; all dogs displayed a pre-test preference for the toy. Kubinyi et al. asked the owners to look at the toy with disgust and at the bracelet with delight. In test trials, both objects were displayed at a short distance, owners directed the dogs to fetch, without giving any directions as to which objects the dogs should fetch. The dogs fetched the toys that they, themselves, preferred—the owners' emotional displays did not “override” the dogs' preferences. In the second study, the objects were presented on a windowsill, out of reach of 51 dogs. In the Toy condition, the owners expressed delight at the toy, matching the dogs' preferences, and in the Bracelet condition, the owners expressed delight at the bracelet. After both objects were placed on the windowsill, the owners commanded the dogs to fetch, again not directing the dogs to either object. Kubinyi et al. measured the dogs' looking times at the objects, finding that, in the matching condition they looked significantly longer at the toy, and there was a trend toward looking longer at the bracelet in the non-matching condition. In a final experiment, with 11 dogs, they found that the dogs were relatively insensitive to owners' direct gaze, suggesting that it was the owners' emotional displays that influenced the dogs' behavior in Study 2.

In another study of dogs' sensitivity to human emotional expressions, Albuquerque et al. presented 52 dogs with a classic detour task, in which a bowl of food was placed behind a V-shaped barrier at the acute angle. In a pre-test phase, the dogs were given the run of the room for 15 s, and six dogs solved the detour task. The study continued with the 46 dogs who failed to find a route around the barrier. The emotional manipulation involved a brief interaction between the demonstrator and the owner that was either positive, negative, or neutral in emotional tone. After this, the test trials began, during which the demonstrator, staying in affective character, baited the bowl in full view of the dogs, thus demonstrating how to circumvent the barrier. Replicating previous research, Albuquerque et al. found that dogs did learn from observing a knowledgeable demonstrator. Contrary to their expectations, however, they also found no influence of the emotional manipulation on dogs' behavior, and they offer several possible explanations for this to inform future research in this area.

Overall, the Research Topic, “Current Perspectives in Cognitive Processing by Domesticated Animals,” provides an exciting overview of a range of recent studies of cognition and behavior in a variety of species. From all studies, new directions for research and insightful new theoretical underpinnings for moving forward in the field of comparative cognition have provided our readership with thoughtful ideas for future research.

## Author Contributions

KB, DL, and SB wrote this Editorial. All authors contributed to the article and approved the submitted version.

## Conflict of Interest

The authors declare that the research was conducted in the absence of any commercial or financial relationships that could be construed as a potential conflict of interest.

## Publisher's Note

All claims expressed in this article are solely those of the authors and do not necessarily represent those of their affiliated organizations, or those of the publisher, the editors and the reviewers. Any product that may be evaluated in this article, or claim that may be made by its manufacturer, is not guaranteed or endorsed by the publisher.

